# NOTIFICATION: The Changing Role of Pathologists from Morphologists to Molecular Pathologists in the Era of Precision Medicine

**DOI:** 10.1155/tbj/9815069

**Published:** 2025-01-13

**Authors:** The Breast Journal

**NOTIFICATION:** S. Masood, “The Changing Role of Pathologists from Morphologists to Molecular Pathologists in the Era of Precision Medicine,” *The Breast Journal* 26, no. 1 (2019): 27–34, https://doi.org/10.1111/tbj.13728.

This notification is for the above article, published online on 25 December 2019 in Wiley Online Library (wileyonlinelibrary.com), and has been issued by agreement between the journal Editor-in-Chief, Dr. Guan-Jun Yang, and John Wiley & Sons Ltd. Following an investigation, the parties learned that an independent peer review process was not conducted for this article prior to publication. The current Editor-in-Chief of the journal, Dr. Guan-Jun Yang, has subsequently reviewed the content of the article and determined that it is suitable to remain published, noting that it does not represent original research, but primarily an editorial contribution from the previous Editor-in-Chief, Dr. Shahla Masood. Therefore, the parties have decided to issue this notification to inform and alert readers.

